# Supplementary material for: Tunable multistate field-free switching and ratchet effect by spin-orbit torque in canted ferrimagnetic alloy
Source: Nat Commun. 2024 Oct 8;15:8713. doi: 10.1038/s41467-024-52834-0 (PMC11461957; doi:10.1038/s41467-024-52834-0)
Supplement: Supplementary file 1 — Supplementary Information [file 41467_2024_52834_MOESM1_ESM.pdf]

Supplementary Information

Tunable multistate field-free switching and ratchet effect by spin-orbit torque in canted ferrimagnetic alloy

Cheng-Hsiang Hsu[1,2*], Miela Gross[3], Hannah Calzi Kleidermacher[1], Shehrin Sayed[1,2], Sayeef Salahuddin[1,2*]

[1]*Department of Electrical Engineering and Computer Science, University of California, Berkeley, California 94720, USA*

[2]*Materials Science Division, Lawrence Berkeley National Laboratory, Berkeley, California 94720, USA*

[3]*Department of Physics, University of California, Berkeley, California 94720, USA*

∗*Correspondence to: C.-H.H. (chhsu@berkeley.edu) or S.S. (sayeef@berkeley.edu).*

This document file includes:

Figure S1-S8

Supplementary Note 1-2

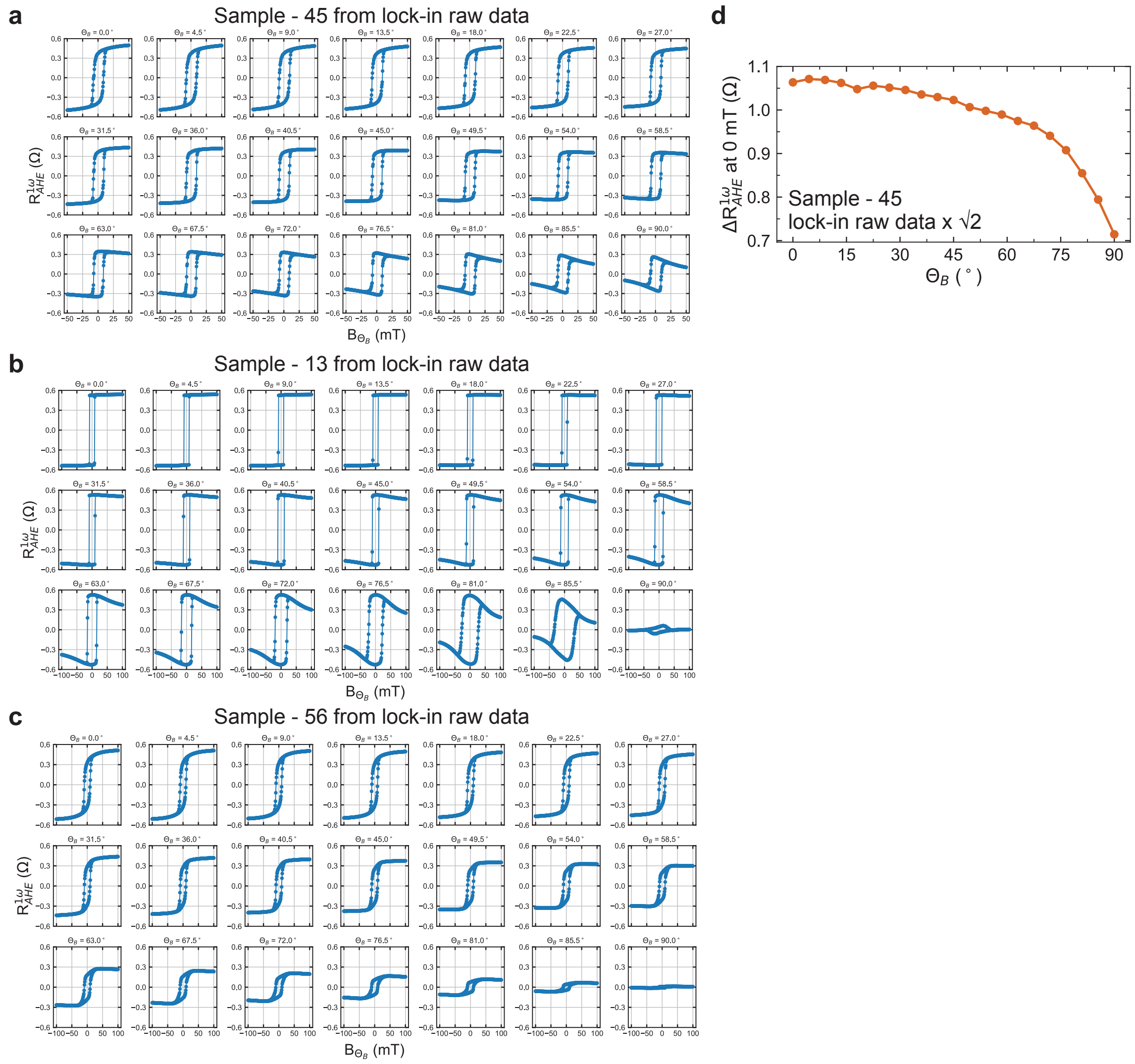

Figure S1. Full data set of angle dependence anomalous Hall effect field scan from 0°(out-of-plane) to 90° (in-plane). Full data set for **(a)** sample-45, **(b)** sample-13, and **(c)** sample-56. $R^{1\omega}_{AHE}$ is obtained by dividing the measured first harmonic voltage by the injected current value. Since the voltage measured through the lock-in amplifier is RMS value, the actual amplitude of the $R^{1\omega}_{AHE}$ can be obtained by multiplying the $R^{1\omega}_{AHE}$ values obtained above by $\sqrt{2}$. **(d)** Actual ΔR_{AHE} amplitude at zero field in sample-45.

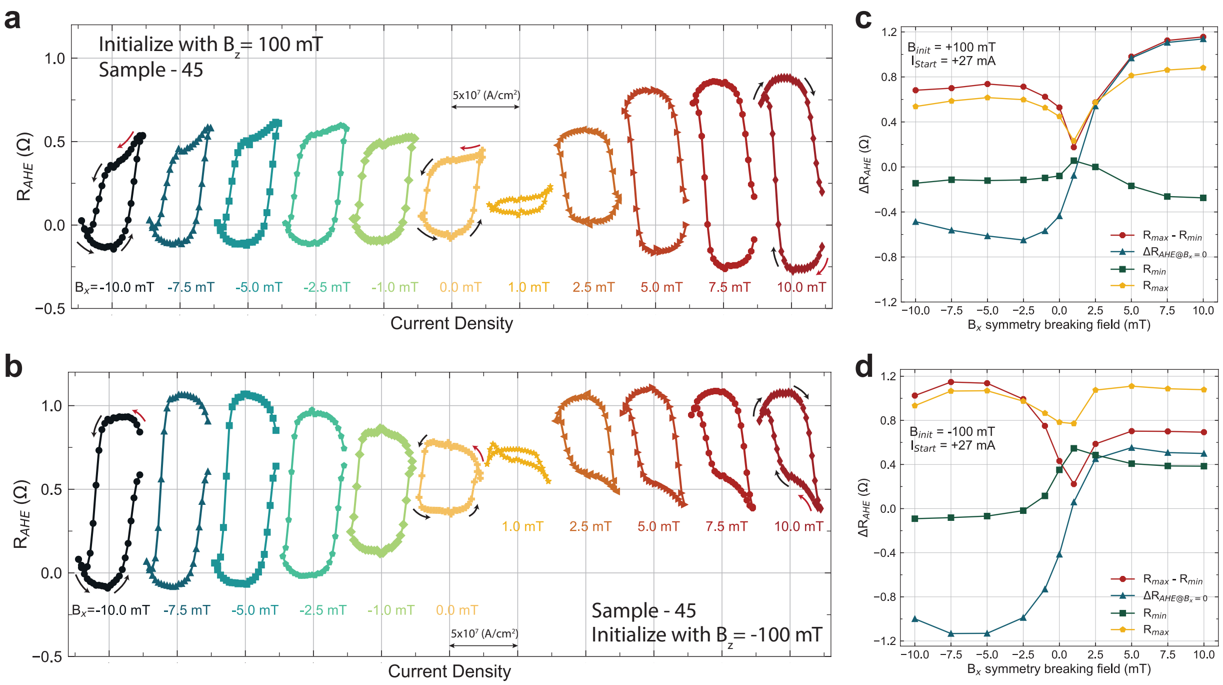

Figure S2. In-plane symmetry breaking field (B_x) dependent switching curves with starting current value of + 27mA and initial set field ($B_{set,z}$) of 100 mT and -100 mT in sample-45. SOT switching curves with varying symmetry breaking field (B_x) from -10 mT to 10 mT. Before each switching experiment, initialization field ($B_{set,z}$) 100 mT and -100 mT was applied to initialize the moments into a known state, respectively. The current pulsing amplitude sequence starts at 27 mA instead of -27 mA in Fig. 4. **(a)** The ΔR_{AHE} for [B_x = 7.5 mT, $B_{z,set}$ = 100 mT] is 1.124 Ohms, which is very similar to the ΔR_{AHE} value (1.064 Ohms from Fig. S1d) from the field sweep AHE hysteresis loop. **(b)** The ΔR_{AHE} for [B_x = -7.5 mT, $B_{z,set}$ = -100 mT] is 1.148 Ohms, which is very similar to the ΔR_{AHE} value (1.064 Ohms) from the field sweep AHE hysteresis loop. **(c)(d)** Extracted information of each switching curve as as function of symmetry breaking field for the case of $B_{set,z}$ = 100 mT and -100 mT respectively. We extracted the ΔR_{AHE}

of maximum and minimum R_{AHE} throughout the entire switching curve, the ΔR_{AHE} of R_{AHE} at the zero current crossing, the minimum R_{AHE}, and the maximum R_{AHE}.

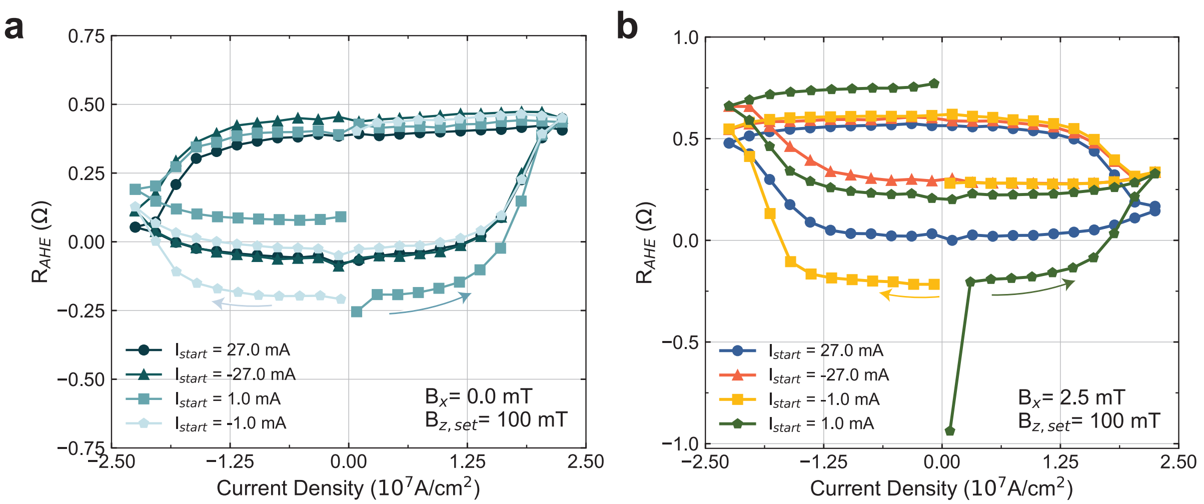

Figure S3. Superposition of spin-orbit torque switching curves with varying starting current amplitude of the pulsing sequence. **(a)** Superposition of the switching curves with varying starting current pulse train amplitude obtained with no symmetry breaking field and initialization field of $B_{z,set}$ = 100 mT. **(b)** Superposition of the switching curves with varying starting current pulse train amplitude obtained with symmetry breaking field B_x = 2.5 mT and initialization field of $B_{z,set}$ = 100 mT. All curves are plotted as measured without normalization, DC shift or any further processing. Multistate behavior can be observed by starting with a small current pulse amplitude and gradually traverse to large current amplitudes to observe the SOT switching. It is also clear that the large initialization $\hat{z}$-field sets the magnetic state to the same state in the curves obtained by starting with a small current amplitude.

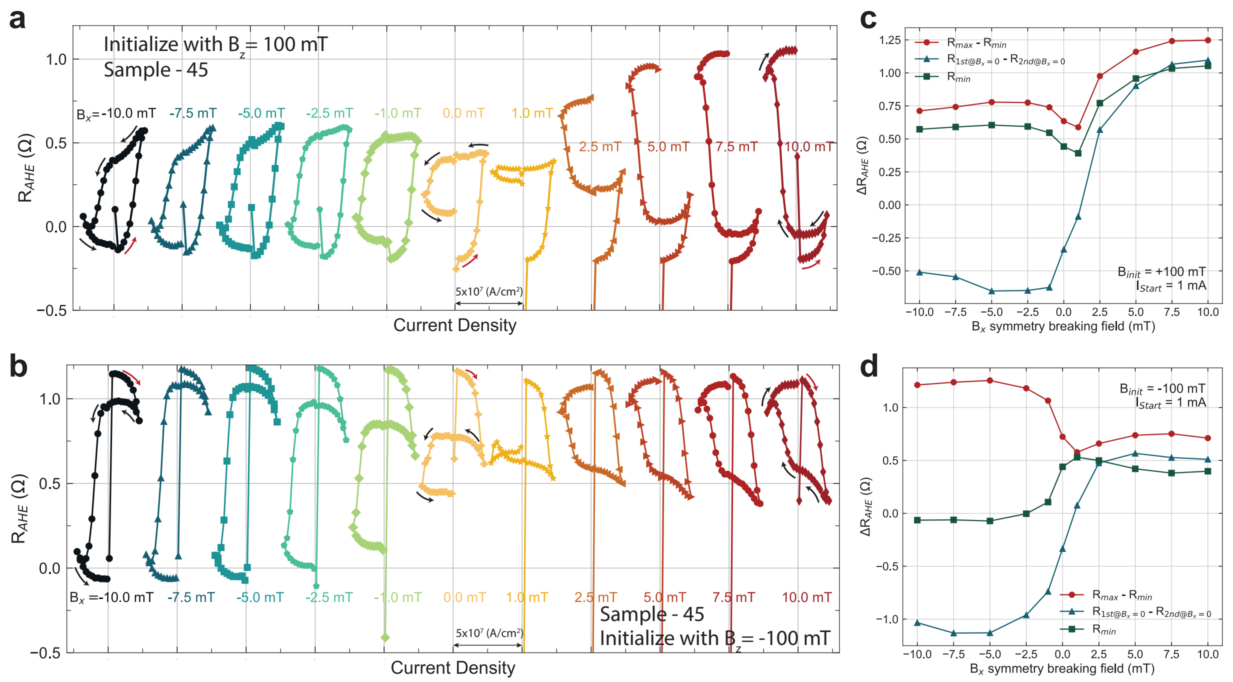

Figure S4. In-plane symmetry breaking field (B_x) dependent switching curves with starting current value of + 1mA and initial set field ($B_{set,z}$) of 100 mT and -100 mT that exhibits the three different switching magnetic states in sample-45. **(a)** The ΔR_{AHE} between the top and middle states for [B_x = 7.5 mT, $B_{z,set}$ = 100 mT] is 1.065 Ohms (from Fig. S1d), which is very similar to the ΔR_{AHE} value (1.064 Ohms) from the field sweep AHE hysteresis loop. The ΔR_{AHE} between the top and bottom states for B_x = 7.5 mT, $B_{z,set}$ = 100 mT] is 1.241 Ohms, which is slightly above the ΔR_{AHE} value (1.064 Ohms) from the field sweep AHE hysteresis loop. **(b)** The ΔR_{AHE} between the top and middle states for [$B_x = -7.5$ mT, $B_{z,set} = -100$ mT] is 1.132 Ohms, which is very similar to the ΔR_{AHE} value (1.064 Ohms) from the field sweep AHE hysteresis loop. The ΔR_{AHE} between the top and bottom states for B_x = 7.5 mT, $B_{z,set}$ = 100 mT] is 1.239 Ohms, which is slightly above the ΔR_{AHE} value (1.064 Ohms) from the field sweep AHE

hysteresis loop. **(c)(d)** Extracted information of each switching curve as as function of symmetry breaking field for the case of $B_{set,z}$ = 100 mT and -100 mT respectively. We extracted the ΔR_{AHE} of maximum and minimum R_{AHE} throughout the entire switching curve, the ΔR_{AHE} of R_{AHE} at the zero current crossing, and the minimum R_{AHE}.

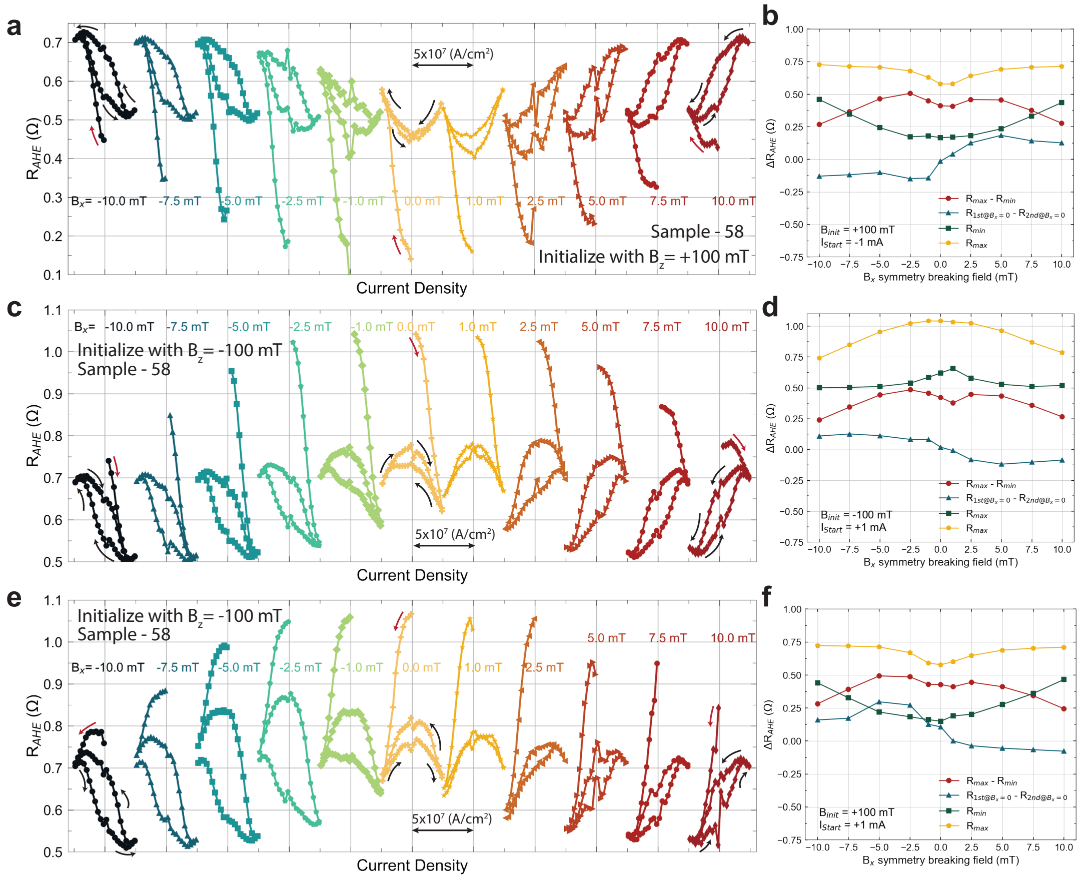

Figure S5. In-plane symmetry breaking field (B_x) dependent switching curves with starting current value of $\pm$ 1mA and initial set field ($B_{set,z}$) of 100 mT and -100 mT that exhibits the three different switching magnetic states in sample-56 with large canting. Switching experiment condition: **(a)** [$B_{set,z}$ = 100 mT, I_{start} = -1 mA] **(c)** [$B_{set,z}$ = -100 mT, I_{start} = 1 mA] **(e)** [$B_{set,z}$ = -100 mT, I_{start} = -1 mA] **(b)(d)(f)** Extracted information of each switching curve as as function of symmetry breaking field for each switching condition. We extracted the ΔR_{AHE} of maximum and minimum R_{AHE} throughout the entire switching curve, the ΔR_{AHE} of R_{AHE} at the zero current crossing, the minimum R_{AHE}, the max R_{AHE} and the min R_{AHE}.

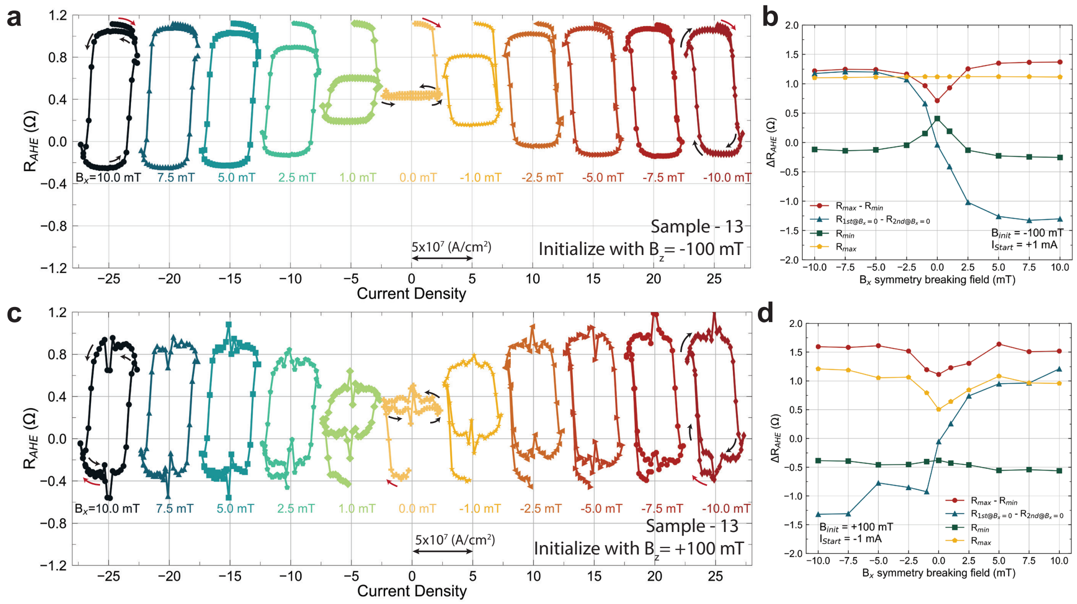

Figure S6. In-plane symmetry breaking field (B_x) dependent switching curves with different starting current amplitude and initial set field ($B_{set,z}$) of 100 mT and -100 mT that exhibits the three different switching magnetic states. **(a)** Switching condition of [I_{start} = 1 mA, $B_{set,z}$ = -100 mT] **(c)** Switching condition of [I_{start} = -1 mA, $B_{set,z}$ = 100 mT] **(b)(d)** Extracted information of each switching curve as as function of symmetry breaking field for each switching condition. We extracted the ΔR_{AHE} of maximum and minimum R_{AHE} throughout the entire switching curve, the ΔR_{AHE} of R_{AHE} at the zero current crossing, the minimum R_{AHE}, the max R_{AHE} and the min R_{AHE}.

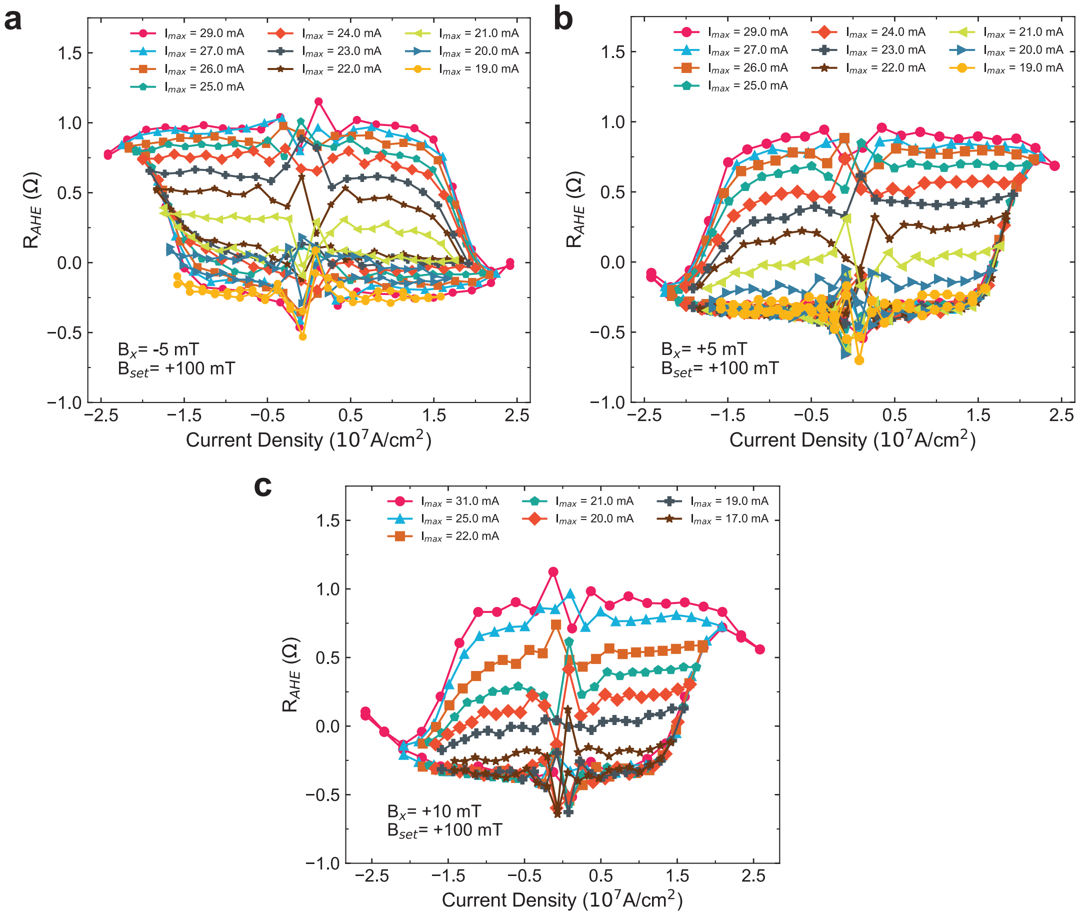

Figure S7. Memristor behavior in sample-13 with small magnetic canting and strong perpendicular magnetic anisotropy. Switching curve as a function of maximum current amplitude in the current pulse train with symmetry breaking field of B_x = **(a)** -5 mT, **(b)** 5 mT, and **(c)** 10 mT are shown. As expected, with a smaller symmetry breaking field, the increment of ΔR_{AHE} is smaller per unit increase in I_{max} by comparing Fig. S7b and S7c. Moreover, the onset of nonzero ΔR_{AHE} is lower with higher symmetry breaking field. In Fig. S7c, switching hysteresis opens up at 17 mA for B_x = 10 mT as compared to 20 mA for B_x = 5 mT.

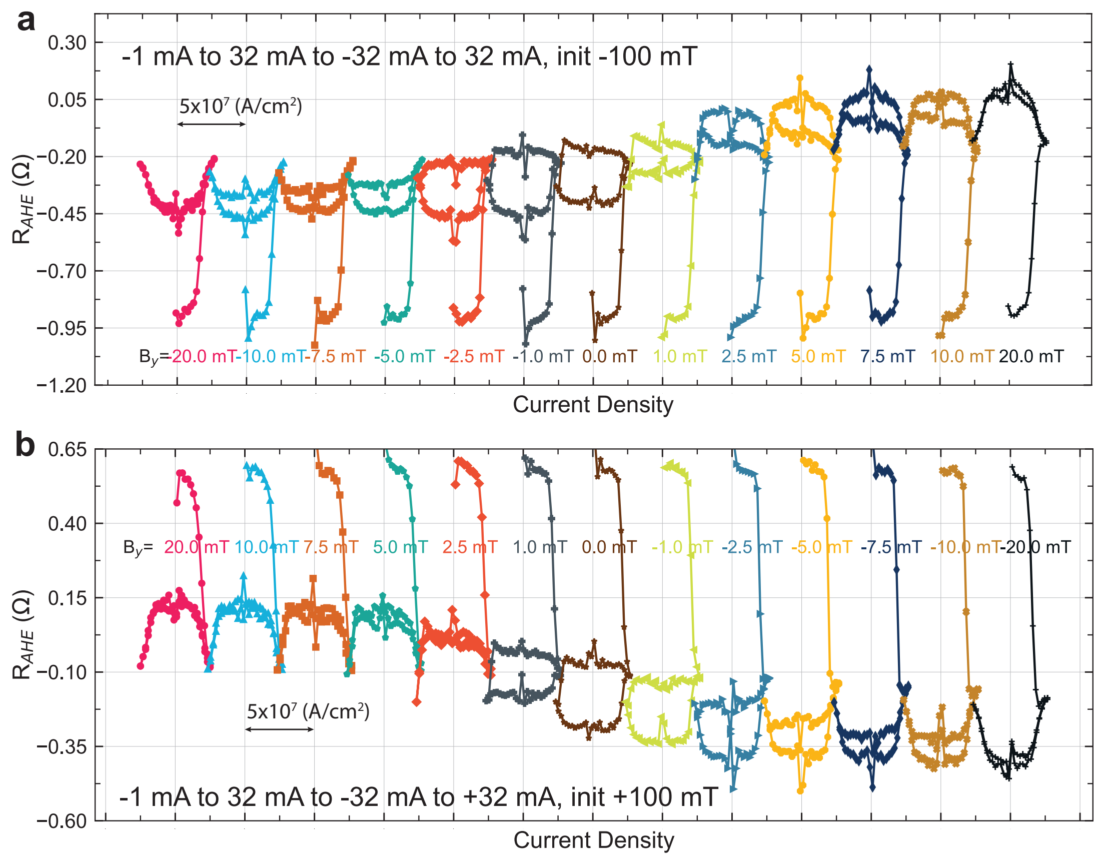

Figure S8. B_y **dependent SOT switching on 13.5° canting device.** SOT switching in a 13.5° canting device with varying B_y IP field applied in the direction (y) transverse to the current direction (x). Device is initialized by **(a)** $B_z = -100$ mT and **(b)** $B_z = 100$ mT.

Supplementary Note 1: Effect of $4f$ shell filling in rare earth elements on the exchange spring behavior in RE-TM ferrimagnetic ultra-thin films

Perpendicular magnetic anisotropy (PMA) in rare earth (RE)-transition metal(TM) ferrimagnetic alloy has been attributed to a growth-induced mechanism[1,2]. In detail, PMA in RE-TM ferrimagnet is mainly due to the pairings of unlike atoms in the out-of-plane direction as compared to the pairings of like atoms in the in-plane direction. As a result, the overlap between the relevant orbitals of the RE atom and TM atoms for ferrimagnetic coupling is very important to induce PMA. In RE-TM ferrimagnetic alloy, the magnetic moment in the RE atom arises from the $4f$ shell whereas the the magnetic moment in the TM atom arises from the $3d$ shell. The antiferromagnetic coupling between the two sublattices are through an indirect exchange interaction between the $4f$ shell of the RE atom and the $3d$ shell of the TM atom through $5d$ shell of the RE atom. Since the $4f$ shell give rise to the magnetism from the RE atom, the filling of the $4f$ shell may be crucial to the magnetic anisotropy. Combining the orbital picture and the growth-induced mechanism, fully filled $4f$ shell in Gd does not provide any directionality as compared to the partially filled $4f$ shell in Tb. Because of this asymmetry in $4f$ shell filling in Tb, PMA has been shown to be more robust in TbCo as compared to GdCo[3]. Although it is more challenging to induce consistent PMA in GdCo, this also means that it is more likely for GdCo to possess canted magnetic anisotropy even though the concentration of GdCo is projected to have PMA. In our study, we break this symmetry by incorporating oxygen into the GdCo alloy which enhances the PMA strength at the atomic level.

Supplementary Note 2: Inducing magnetic canting via oxidation in GdCo heterostructure

The oxygen plasma treatment to induce the magnetic canting is a very gentle oxygen plasma process typically designed for descum of photoresist residue in cleanroom fabrication process. The parameters are 50 W RF power, 50°C sample chuck, and 180 mTorr oxygen pressure. They key to obtain the canting is to perform such oxidation process on a device fabricated from GdCo heterostructure thin film that has composition close to the magnetic compensation. As one increases the oxidation duration, the PMA will enhance but stops beyond a certain limit and even degrades the PMA. At this point, the oxygen content is likely saturated near the top of the Pt/GdCo/Ta heterostructure thus no longer enhancing the PMA. Then, one will need to let the sample stay under ambient condition (room temperature and atmospheric pressure). This step is to allow the oxygen to diffuse into the bulk of GdCo. After a day, the PMA strength will increase significantly. This process should theoretically be accelerated by annealing the device, however, we did not attempt to optimize the process in this study. One can see that in Fig. Ox of the main text, the PMA enhances significantly after 17 hours from the 210 s of oxidation. However, by oxidizing the device by an additional 20 s (total 230 s), the PMA is degraded. As a result, both the oxidation and the diffusion of the oxygen are crucial steps in inducing PMA thus the canting in such GdCo heterostructure.

References

1. Hellman, F. & Gyorgy, E. M. Growth-induced magnetic anisotropy in amorphous Tb-Fe. *Physical Review Letters* **68**, 1391–1394 (1992).

2. Harris, V. G., Aylesworth, K. D., Das, B. N., Elam, W. T. & Koon, N. C. Structural origins of magnetic anisotropy in sputtered amorphous Tb-Fe films. *Physical Review Letters* **69**, 1939–1942 (1992).

3. Ceballos-Sanchez, A. *Characterization and Manipulation of Temperature-Driven Magnetic Phenomena: Magnetic Transition in FeRh Thin Films and Ultrafast Magnetization Reversal in a-Gd22-xTbxCo78 Thin Films*. Ph.D. thesis, University of California, Berkeley (2019).
